# Prognostic Evidence of the miRNA-Based Ovarian Cancer Signature MiROvaR in Independent Datasets

**DOI:** 10.3390/cancers13071544

**Published:** 2021-03-27

**Authors:** Loris De Cecco, Marina Bagnoli, Paolo Chiodini, Sandro Pignata, Delia Mezzanzanica

**Affiliations:** 1 Integrated Biology Platform, Department of Applied Research and Technology Development, Fondazione IRCCS Istituto Nazionale dei Tumori, 20133 Milan, Italy; 2 Molecular Therapies Unit, Department of Research, Fondazione IRCCS Istituto Nazionale dei Tumori, 20133 Milan, Italy; marina.bagnoli@istitutotumori.mi.it; 3 Medical Statistics Unit, University of Campania “Luigi Vanvitelli”, 80138 Naples, Italy; paolo.chiodini@unicampania.it; 4 Urogynaecological Medical Oncology Unit, Istituto Nazionale Tumori–IRCCS-“Fondazione G. Pascale”, 80131 Naples, Italy; s.pignata@istitutotumori.na.it

**Keywords:** epithelial ovarian cancer, microRNA, molecular predictor, early relapse, independent validation

## Abstract

**Simple Summary:**

Epithelial ovarian cancers (EOC) have an unpredictable frequent recurrence often associated with incurable chemo-resistant disease. Basing on the miRNA expression profile of 892 EOC patients, we previously developed a 35 miRNA-based classifier, MiROvaR, able to predict EOC risk of early relapse. Further independent analysis of prediction accuracy represents a crucial step in the test-validation phase. Here we exploited an external and independently collected, handled and profiled EOC cohort, to challenge MirovaR accuracy. Our analysis confirmed the MiROvaR prognostic power, thus opening the way to its prospective validation as a clinical grade assay entering into clinical practice to help in the refinement of therapeutic intervention for high risk EOC patients.

**Abstract:**

Epithelial ovarian cancer (EOC) remains the second most common cause of gynecological cancer deaths. To improve patients’ outcomes, we still need reliable biomarkers of early relapse, of which external independent validation is a crucial process. Our previously established prognostic signature, MiROvaR, including 35 microRNAs (miRNA) able to stratify EOC patients for their risk of relapse, was challenged on a new independent cohort of 197 EOC patients included in the Pelvic Mass Study whose miRNA profile was made publically available, thus resulting in the only accessible database aside from the EOC TCGA collection. Following accurate data matrix adjustment to account for the use of different miRNA platforms, MiROvaR confirmed its ability to discriminate early relapsing patients. The model’s original cutoff separated 156 (79.2%) high- and 41 (20.8%) low-risk patients with median progression free survival (PFS) of 16.3 months and not yet reached (NYR), respectively (hazard ratio (HR): 2.42–95% Confidence Interval (CI) 1.49–3.93; Log-rank *p* = 0.00024). The MiROvaR predictive accuracy (area under the curve (AUC) = 0.68; 95% Cl 0.57–0.79) confirms its prognostic value. This external validation in a totally independently collected, handled and profiled EOC cohort suggests that MiROvaR is a strong and reliable biomarker of EOC early relapse, warranting prospective validation.

## 1. Introduction

Epithelial ovarian cancer (EOC) is a life-threatening disease characterized by late-stage presentation, high pathological and molecular heterogeneity and frequent progression to an incurable state of chemo-resistant recurrent disease [1].

In the last 10 years, great efforts have been made to better characterize EOC to improve patients’ stratification. Broad international, collaborative studies have been performed primarily on high-grade serous EOC (HGSOC) to derive transcriptome-based molecular subtypes to guide patients’ management. However, the great genomic and spatial heterogeneity of HGSOCs posed a serious limitation to their clinical application. Yet, the recently defined ProTYPE [2] HGSOC molecular classifier, although it has reached the clinical grade level, still needs prospective validation without clear prognostic potential. In fact, with the exception of BRCA1/2 mutations guiding the use of PARP inhibitors [3], no transcriptome-based molecular classifiers have entered clinical practice.

The identification of high-risk EOC patients still remains an urgent clinical need to improve the design of tailored therapy. By relying on the master layer of regulation for gene expression provided by microRNAs (miRNAs) [4], we identified a 35 miRNA-based molecular predictor—MiROvaR—able to stratify EOC patients for their risk of relapse independently from the two strongest prognostic clinical variables so far available for EOC: the International federation of Gynecology and Obstetrics (FIGO) stage and residual disease after primary surgery [5]. The molecular predictor was developed by profiling 179 patients from the Multicenter Italian Trials in Ovarian Cancer (MITO)2 clinical trial [6], and validated in two independent cohorts: the TCGA collection of EOC [7] and a set of patients from our laboratory. We then demonstrated its ability to encompass the biological and molecular differences among the histological subtypes of EOC.

Here, we challenge MiROvaR performance in external and independent EOC Danish case material profiled for the identification of prognostic/predictive miRNA [8].

## 2. Materials and Methods

### 2.1. Data Processing

A total of 197 formalin-fixed, paraffin-embedded specimens from patients with EOC belonging to the “Pelvic Mass Study,” recruited at the Gynecologic Department, Rigshospitalet (Copenhagen University Hospital, Denmark), were profiled for microRNA expression [8] using GeneChip 1.0 miRNA microarrays (Affymetrix) that allowed the detection of 847 different human miRNAs. Normalized data were retrieved from NCBI’s Gene Expression Omnibus (GEO) repository under the accession number GSE94320. To account for the different miRNA platforms used, Affymetrix in the “Pelvic Mass Study” [8] and Agilent/Illumina in our paper [5], Prahm’s data [8] were processed using the justRMA function [9] to apply the RMA procedure. The intensity measures were background adjusted and normalized by the quantile method, with resulting expression levels on a log2-based scale [10]. Based on the 385 miRNAs shared among platforms in our previous work [5], 382 (99.2%) were present in the Affymetrix arrays. To assess MiROvaR on GSE94320, this data matrix and those used by Bagnoli et al. [5] were adjusted to reduce the likelihood of systemic, non-biological, technical experimental biases by ComBat algorithm [11].

The rescaled data distribution, resulting in skewness of −0.756 and kurtosis of 2.93, enabled the application of the MiROvaR model with threshold for patients’ stratification in risk classes, as defined in Bagnoli et al. [5]. Skewness is a measure of the symmetry of the data distribution, while kurtosis measures the tail-heaviness of the distribution. Skewness and kurtosis were computed by D’Agostino and Anscombe-Glynn tests, respectively [12].

### 2.2. Statistical Analysis

The clinical endpoint of the study is progression-free survival (PFS), defined as the time from primary surgery until relapse, progressive disease or death of any cause, whichever occurred first. All clinical data were retrieved from Prahm’s paper [8].

For biomarker optimal cutoff determination, we used the Cutoff Finder R package available at http://molpath.charite.de/cutoff (accessed on 15 January 2021) [13], with optimization of the correlation with PFS data; in this case, the survival analysis was performed using the coxph and survfit functions of the survival R package [14]. A Cox proportional hazard model was fitted to the dichotomized variable and the survival data. The point with the most significant (log-rank test) split was considered as being the optimal cutoff. Hazard ratios (HRs) including 95% confidence intervals were calculated and plotted in the range of biomarker expression.

To assess the overall performance of our model, the prediction error [15] was evaluated, defined through Brier’s score as a function of survival time and computed using the R package pec [16]. The prediction error established the relative worth of prediction risk obtained by our signature over time, and was computed by including our miRNA model and fitting a likelihood Cox-proportional hazard model. With this tool, the predicted risks of progression are grouped according to the deciles of their distribution, and for each decile, the observed proportion of an event is plotted against the mean value predicted by the miRNA model. The null model corresponds to a model wherein no data on the covariates are used. To evaluate the prediction error curves, the miRNA model and the null model are plotted over time. The benchmark value of 0.25 reached by the null model corresponds to a 50% risk prediction probability for every sample. The leave-one-out cross validation estimate of the prediction error was calculated for all event times, using a time-dependent adaption of the Brier score.

To evaluate a prediction model that gives a continuous range of probabilities, discrimination and calibration are recommended [17]. Discrimination was evaluated by time-dependent receiver operating curve (ROC) [18], computed using the timeROC R package [19]. With the information of follow-up and PFS, the AUC can be computed at several time points, and MirOvaR prognostic ability can be evaluated over time.

All tests were two-sided with a *p*-value considered significant at <0.05, and were performed using R software, version 3.6.0 (http://www.r-project.org/ accessed on 15 January 2021).

## 3. Results and Discussion

The Danish case material described by Prahm et al. [8] includes 197 EOC patients, representative of EOC demography and incidence, with a median follow-up time of 88 months, a median progression-free survival (PFS) of 19 months and a median overall survival of 49 months (clinical-pathological characteristics have been already detailed [8] and summarized in Table S1). As reported by Prahm et al. [8], the patients’ classification according to MiROvaR prognostic risk index, using the original cutoff defined in our paper [5], was inapplicable due to differences in miRNA dynamic ranges across the microarray platforms used. However, as suggested by the authors [8], when the median MiROvaR value was imposed as a cutoff, a significant prognostic stratification of EOC according to their risk of relapse was obtained (Figure S1).

**Figure 1:**
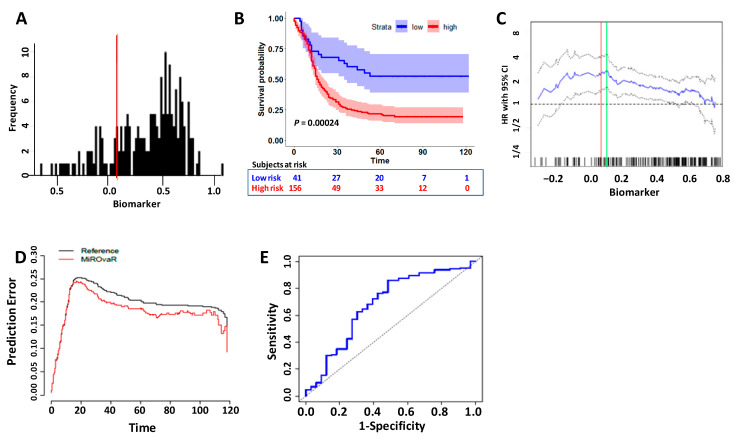
Progression-free survival (PFS) of epithelial ovarian cancers (EOC) patients in Prahm’s dataset (GSE94320) stratified by risk according to MiROvaR model. (**A**). MiROvaR index. Affymetrix microarray data were retrieved from Gene Expression Omnibus (GEO) repository and MiROvaR index was computed in GSE94320 after adjustment to account for the microarray platforms. The bar-plot depicts the MiROvaR index showing skewness = −0.756 and kurtosis = 2.93. The red bar shows the model cutoff value (=0.07359) as determined in our original paper and used in the present analysis. (**B**). Kaplan–Meier curves according to the MiROvaR value as cutoff: blue and red lines indicate low- and high-risk patients reaching HR = 1.42 (CI 1.49–3.93), *p* = 0.00024. High- and low-risk curves were compared with the long-rank test. HR = hazard ratio. Shadows indicate upper and lower 95% confidence intervals. (**C**). Hazard ratio assessed with PFS as the endpoint and independent of the cutoff point for the MiROvaR index. The red bar shows the model cutoff value (=0.07359), while the green bar designates the optimal cutoff point (=0.1085) with the most significant HR split. Solid and broken lines indicate the HR and the 95% confidence intervals. (**D**) Curves of time-dependent prediction errors by Brier scores to evaluate MiROvaR performance for predicting PFS in EOC. The Brier score for our model (red line) is computed along with the reference (i.e., the marginal Kaplan–Meier estimator, ignoring the predictors). (**E**). Time-dependent receiver operating curve (ROC) in Prahm’s dataset for MirOvaR predicting 72-month time point. The AUC is = 0.68 (95% CI 0.57–0.79).

To challenge the MiROvaR model on this independent case material, data had to be adjusted taking into account the different methodologies used in Prahm’s [8] and our [5] studies, which were based on Affymetrix and Agilent/Illumina platforms, respectively. Essentially, following background subtraction and normalization, 382 (99.2%) of the 385 miRNAs shared among our Agilent/Illumina platforms were also detected in the Affymetrix arrays. The data were further adjusted to reduce the likelihood of systemic, non-biological, technical experimental biases by the ComBat algorithm [9]. All the 35 miRNAs belonging to MiROvaR signature were detected on the Affymetrix arrays. The rescaled data distribution allowed for the assessment of the prognostic potential of MiROvaR by imposing the original model cutoff (0.07359) that stratified the patients into 156 (79.2%) high- and 41 (20.8%) low-risk (Figure 1A) categories. The model separated high- and low-risk patients with a median PFS of 16.3 months and not yet reached (NYR), respectively (HR: 2.42–95% CI 1.49–3.93; Log-rank *p* = 0.00024) (Figure 1B). Moreover, when the MiROvaR index was plotted against HR, the optimal cutoff value (the cutoff point with the most significant HR as determined by applying the Cutoff Finder tool) [10] corresponded to 0.1085, close to the published model cutoff (Figure 1C). With the optimal cutoff, only a marginal improvement was observed as compared to the original model cutoff (see Figure 1B); in fact, the best cutoff separated 152 (78.2%) high- and 45 (22.8%) low-risk patients (Figure S2A), with median progression-free survival (PFS) of 16 months and not yet reached (NYR), respectively (HR: 2.65–95% Cl 1.65–4.27; Log-rank *p* = 0.00003) (Figure S2B).

The traditional approach to assess the performance of a prognostic model in independent external datasets is to quantify how close predictions are to the actual outcome [20], assessing measures of overall performance and measures of discrimination (sensitivity and specificity). For overall performance, we evaluated the prediction error through the Brier score. As shown in Figure 1D, the score for MiROvaR (red line) is lower than the null model, denoting superior model performance over the period of follow-up [21]. The accuracy of the model was assessed by generation of the ROC curve and evaluation of AUC that reaches a value of 0.68; 95% CI 0.57–0.79 (Figure 1E). For the sake of completeness, a time-dependent ROC curve was computed measuring the performance of our model over time (Figure S3).

Our primary aim was to validate the performance of our miRNA-based molecular predictor of EOC early relapse. Extending our analysis to clinically relevant covariates available in Prahm’s dataset, a significant association of MiROvaR high risk was observed with serous histotype, high grade, and the presence of residual disease at primary surgery (Table 1). MiROvaR is intended to be a widely useful tool that could encompass the biological/molecular differences among the histological sub-types of EOC. However, we confirmed its validity in a homogeneous sub-group of HGSOC (from the TCGA dataset and from the second validation set used in our original paper [5]). When considering HGSOC only in the Danish case material, the proportion of low-risk HGSOC was too low to have a statistically significant impact; however, the median PFS for the MiROvaR low-risk group was 36 months (50% recurrence rate) versus 15 months (83% recurrence rate) for the MiROvaR high-risk group.

Although in the Danish case material the categorization of the patient population for FIGO stage was described (Table S1), this clinical parameter was not associated with the single-patient molecular profile, and therefore it cannot be included into the association analysis (see Table 1).

The lack of annotation of FIGO stage among the characteristics included into the matrix of molecular data effected also the possibility of assessing the prognostic independence of MiROvaR, adjusting for the clinical parameters that we used in our former study [5], i.e., residual disease and FIGO stage. Eventually, in a bivariate analysis adjusting for residual disease (the only prognostic clinical covariate here available), MiROvaR confirmed its independent prognostic value (Table 2).

The proposed categorization for residual disease (suboptimal vs. optimal) has been included into the analysis, since the training set used for MiROvaR development was derived from a MITO trial [6] that used this categorization. However, we clearly showed that in the three independent multicenter case materials used for its development and totally accounting for over 890 patients, MiROvaR also performed with the residual disease categorized as NED (not evident disease) vs. <1 cm vs. >1 cm [5]. A post-hoc analysis performed categorizing those patients according to current guidelines for residual disease (presence of residual vs. NED) again confirmed MiROvaR independency. In the Prahm case material, when adopting this classification, we observed a significant interaction between MiROvaR and residual disease (*p* = 0.016). However, it is worth noting that in patients with no residual disease, MiROvaR significantly and efficiently discriminated those with worse prognosis (HR 2.70; 95%CI 1.26–5.79; *p*-value 0.011). This can clearly have clinical relevance, since those patients—according to a prognostic stratification based on residual disease and without a further molecular classification—would all have been considered as exhibiting good prognosis.

The management of ovarian cancer is evolving from a “one-size-fits-all” approach to more precise interventions that take into account the tumors’ molecular characteristics [22]. These tailored approaches need the discovery of prognostic/predictive biomarkers guiding the selection of patients who are most likely to benefit from further therapeutic interventions, or a de-escalation to reduce unnecessary toxicity. While a clinical-grade assay for the transcriptome-based classification of HGSOC histotype is ready for “the prime time” [2], the new knowledge derived by single cell sequencing approaches adds a further level of complexity that suggests a re-interpretation, and actually limits the efficacy of transcriptome-based discrete HGSOC subtyping [23]. We have shown the potential role of miRNAs in predicting disease progression [5,24], and the Prahm et al. study [8] enabled us to test our MiROvaR model in a totally independently collected, handled and profiled EOC patient dataset, thus following the rules for biomarkers validation [25], and confirming MiROvaR reliability as a biomarker of EOC early relapse, regardless of their histotypes. A biomarker such as MiROvaR, based on a tumor’s molecular characteristics at diagnosis, once validated also on liquid biopsy, might help in patients’ selection before any therapeutic treatment. Furthermore, by giving information on the biological characteristics of the tumor, MiROvaR may help in identifying new actionable targets. From a clinical point of view, the subgroup of patients with unfavorable prognosis identified by MiROvaR might be a candidate for more aggressive treatment modalities. In the late stage, setting MiROvaR may help in classifying HR-proficient patients at high risk of relapse who could benefit from Bevacizubam treatment/maintenance rather than PARP inhibitors. In the early stage setting, MiROvaR could help in identifying a group of patients at higher risk of relapse, who would really benefit from the adjuvant chemotherapy, and a group of patients at lower risk for whom unnecessary treatments can be avoided.

## 4. Conclusions

At a methodological level, we should highlight the importance of publically available genomics data with associated, well-controlled clinical data to enable their wise reuse with new bioinformatic tools. Furthermore, at the clinical level, we believe that the herein presented data represent an important step forward in establishing the role of miRNAs as biomarkers in ovarian cancer, thus warranting a prospective validation hopefully resulting in MiROvaR entering into clinical practice.

## Figures and Tables

**Table 1:** Distribution of MiROvaR high- and low-risk patients from the Danish case material in relation to clinical and pathological variables.

Clinical Characteristics	Low Risk		High Risk		*p* Value
	N	%	N	%	
*Age, years*					
Median	60		66		
Range	31–81		31–89		
*Histology*					<0.0001
Serous	22	14	140	86	
Endometroid	7	47	8	53	
Mucinous	8	73	3	27	
Clear Cells	4	44	5	56	
*Grade*					<0.0001
1, well differentiated	11	55	9	45	
2, moderately differentiated	22	22	80	78	
3, poorly differentiated	8	11	66	89	
Missing information			1		
*Surgical debulking*					0.002
Optimal (<1 cm)	35	28	91	72	
Suboptimal (>1 cm)	6	8	65	92	

**Table 2:** Cox proportional hazard regression analysis.

Covariates	Univariate Analysis		Multivariate Analysis	
	HR (95% CI)	*p*-Value	HR (95% CI)	*p*-Value
MiROvaR (high- vs. low-risk)	2.42 (1.49–3.93)	0.000367	1.75 (1.1–2.89)	0.0282
Residual disease (suboptimal vs. optimal)	4.28 (3–6.1)	<0.0001	3.82 (2.65–5.49)	<0.0001

HR, Hazard ratio; CI, confidence interval.

## Data Availability

Normalized data for the reported analysis were retrieved from NCBI’s Gene Expression Omnibus (GEO) repository under the accession number GSE94320.

## Supplementary Materials

The following are available online at https://www.mdpi.com/article/10.3390/cancers13071544/s1. Figure S1: Progression-free survival of EOC patients in Prahm’s dataset1 (GSE94320) stratified by risk according to MiROvaR median cutoff, Figure S2: Progression-free survival of EOC patients in Prahm’s dataset 1 (GSE94320) stratified by risk according to MiROvaR best cutoff, Figure S3: MiROvaR predictive accuracy over-time, Table S1: Clinical and pathological characteristics of patients from the Danish case material.
