# Supplementary material for: Prognostic Evidence of the miRNA-Based Ovarian Cancer Signature MiROvaR in Independent Datasets
Source: Cancers (Basel). 2021 Mar 27;13(7):1544. doi: 10.3390/cancers13071544 (PMC8037414; doi:10.3390/cancers13071544)
Supplement: Supplementary file 1 [file cancers-13-01544-s001.pdf]

cancers MDPI

Supplementary Materials: Prognostic Evidence of the miRNA-Based Ovarian Cancer Signature MiROvaR in Independent Datasets

Loris De Cecco, Marina Bagnoli, Paolo Chiodini, Sandro Pignata and Delia Mezzanzanica

Table S1. Clinical and pathological characteristics of patients from the Danish case material.

Clinical characteristics	N° (197)	%
**Age, years**		
Median	64	
range	31–89	
**Histology**		
Serous	162	82
Endometroid	15	8
Mucinous	11	6
Clear Cells	9	5
**Stage (FIGO)**		
I	31	16
II	21	11
III	119	60
IV	26	13
**Grade**		
1, well differentiated	20	10
2, moderately differentiated	102	52
3, poorly differentiated	74	38
Missing information	1	<1
**Amount of residual disease**		
NED	94	48
<1 cm, mRD	32	16
>1 cm, GRD	71	36
**Median follow up (months)**	88 (61-126)	

FIGO=International federation of Gynecology and Obstetrics; NED = not evident disease; mRD = minimal residual disease; GRD=gross residual disease.

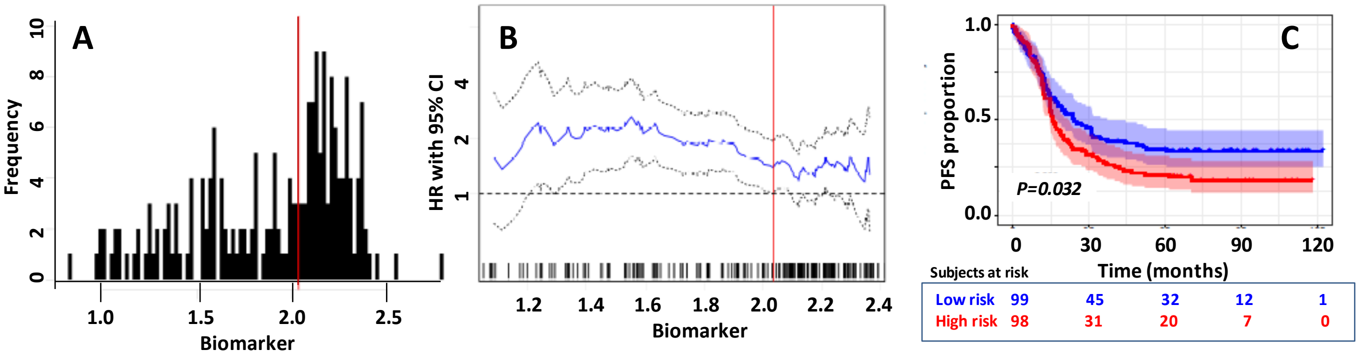

Figure S1. Progression-free survival of EOC patients in Prahm's dataset1 (GSE94320) stratified by risk according to MiROvaR median cutoff. (**A**) MiROvaR index. Affymetrix microarray data were retrieved from GEO repository. The barplot depicts the MiROvaR index in the original Prahm's dataset before adjustment with skewness = −0.663 and kurtosi s= 2.49; the red bar shows the median value (=2.036). (**B**) Hazard ratio assessed with PFS as endpoint and independent of the cutoff point for the MiROvaR index. The vertical red line denotes the median index value. Solid and broken lines indicate the HR and the 95% confidence intervals. (**C**) Kaplan–Meier curves according to the median MiROvaR value as cutoff (before adjustment): blue and red lines indicate low- and high-risk patients reaching median PFS of 24.3 and 15.9 months, respectively, with HR = 1.41 (CI 1.01–1.96), $p = 0.032$. High- and low-risk curves were compared with the long-rank test. HR=hazard ratio. Shadows indicate upper and lower 95% confidence intervals.

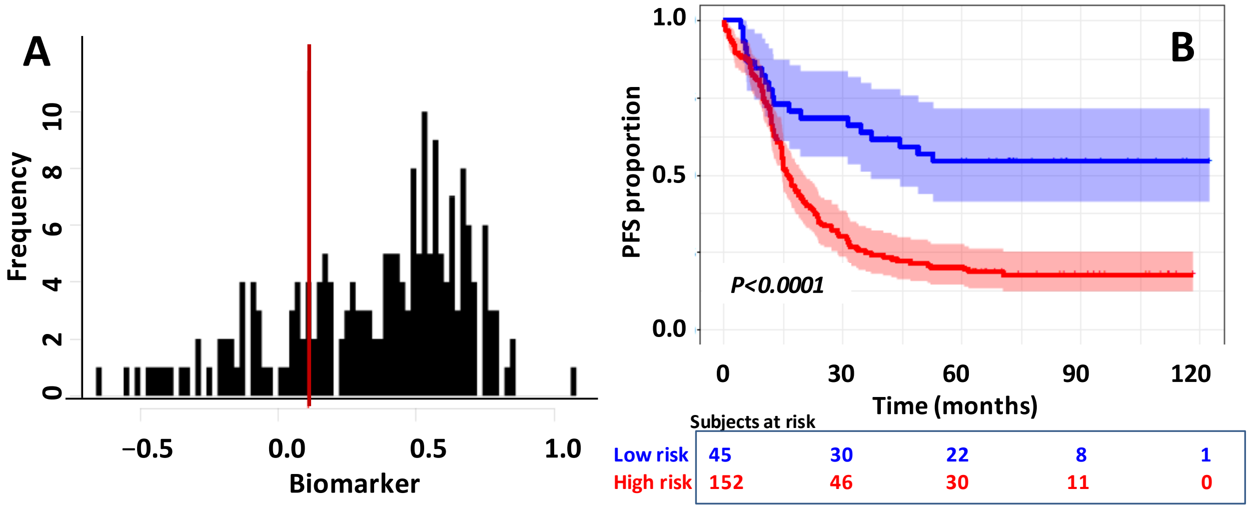

Figure S2. Progression-free survival of EOC patients in Prahm's dataset 1 (GSE94320) stratified by risk according to MiROvaR best cutoff. (**A**) MiROvaR index. MiROvaR index was computed in GSE94320 after adjustment to account for the microarray platforms. The red bar shows the best cutoff value (=0.01085). (B) Kaplan–Meier curves according to the MiROvaR best cutoff value: blue and red lines indicate low- and high-risk patients reaching median PFS of 16.2 and NYR months, respectively, with HR = 2.65 (CI 1.65-4.27), $p = 0.00003$. High- and low-risk curves were compared with the long-rank test. HR = hazard ratio. Shadows indicate upper and lower 95% confidence intervals.

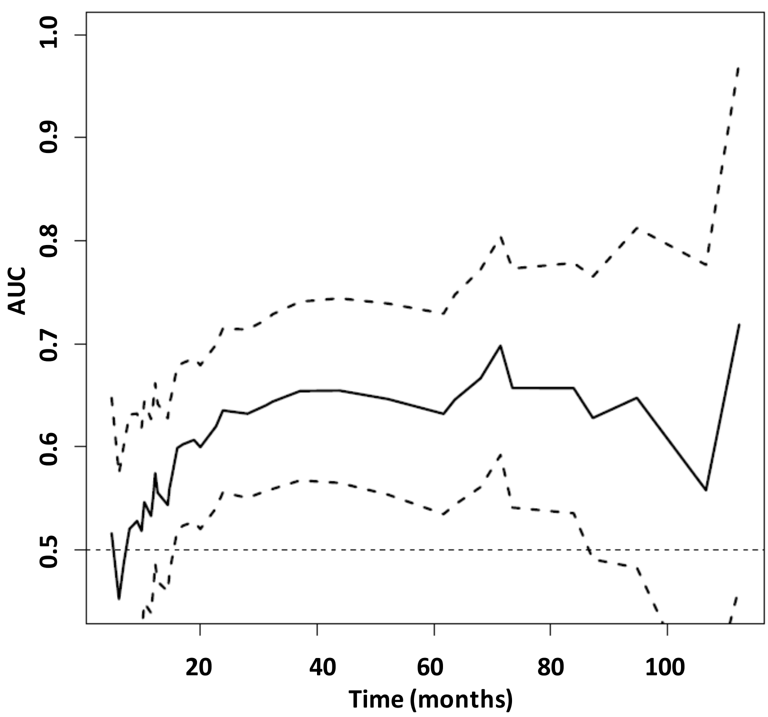

Figure S3. MiROvaR predictive accuracy over-time. Time-dependent receiver operating curve (ROC) analysis showing AUCs for MiROvaR and PFS over follow-up time.
